# P-2138. Evaluation of Risk Factors for Neurological Dysfunction in Hospitalized Patients Receiving Cefepime or Piperacillin/Tazobactam

**DOI:** 10.1093/ofid/ofaf695.2301

**Published:** 2026-01-11

**Authors:** Daniel Scozzari, Michael E DeWitt, Christopher Buckley, Katelyn Jimison, Alaina Shukdinas, John C Williamson

**Affiliations:** Atrium Health Wake Forest Baptist - High Point Medical Center, High Point, North Carolina; Atrium Wake Forest Baptist Health/ Wake Forest University School of Medicine, Winston-Salem, North Carolina; Vanderbilt University Medical Center, Nashville, Tennessee; Atrium Health Wake Forest Baptist Medical Center, Winston-Salem, North Carolina; Atrium Health Wake Forest Baptist Medical Center, Winston-Salem, North Carolina; Wake Forest School of Medicine, Winston-Salem, NC

## Abstract

**Background:**

Beta-lactam antibiotics are associated with risk for neurotoxicity. Cefepime (FEP) may be more likely to cause neurological dysfunction (ND) due to its ability to cross the blood brain barrier and antagonize GABA receptors. In a secondary analysis, a recent randomized trial of FEP vs piperacillin/tazobactam (P/T) found a relatively higher rate of ND among patients receiving FEP. We sought to determine what factors, including antibiotic choice, may be associated with ND among patients receiving FEP or P/T.

**Figure:**
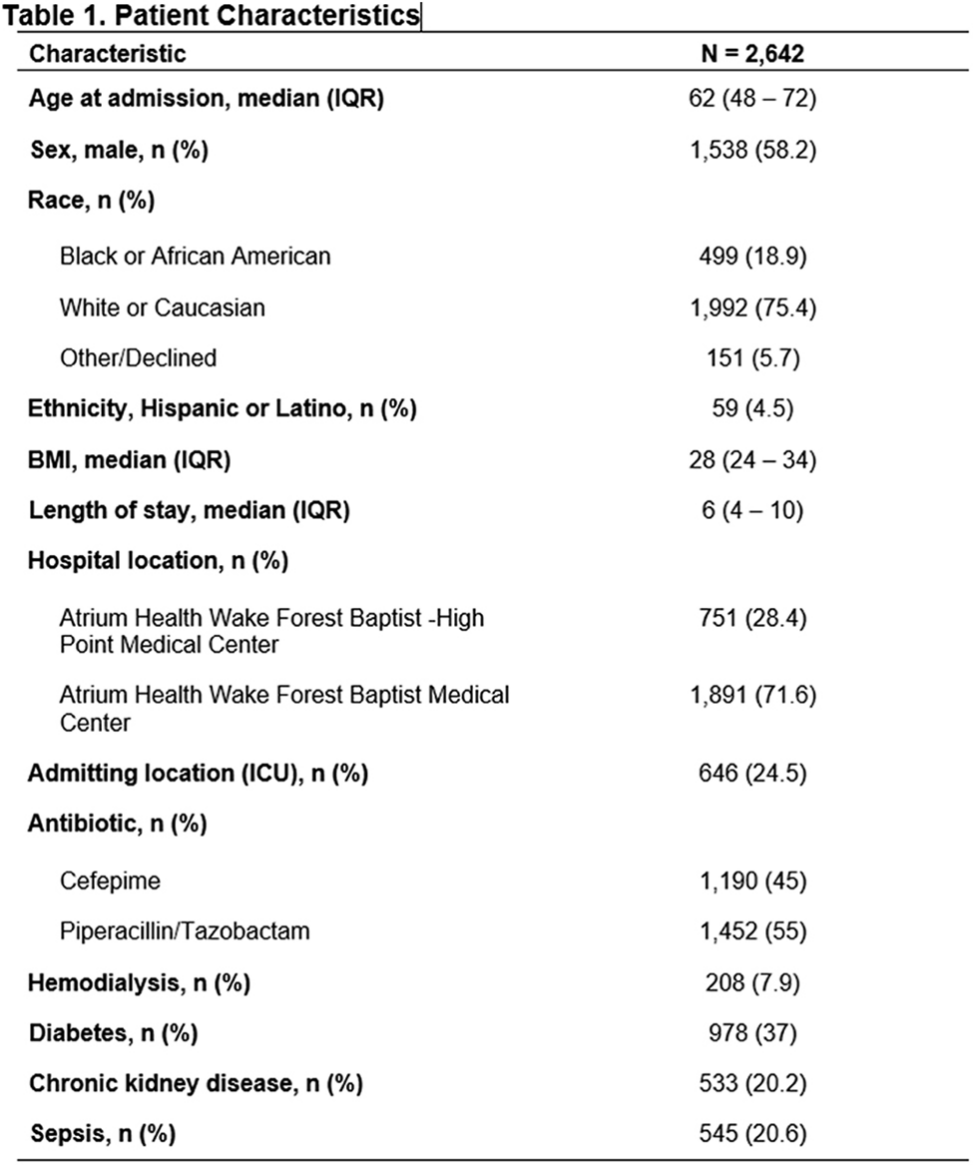

**Figure:**
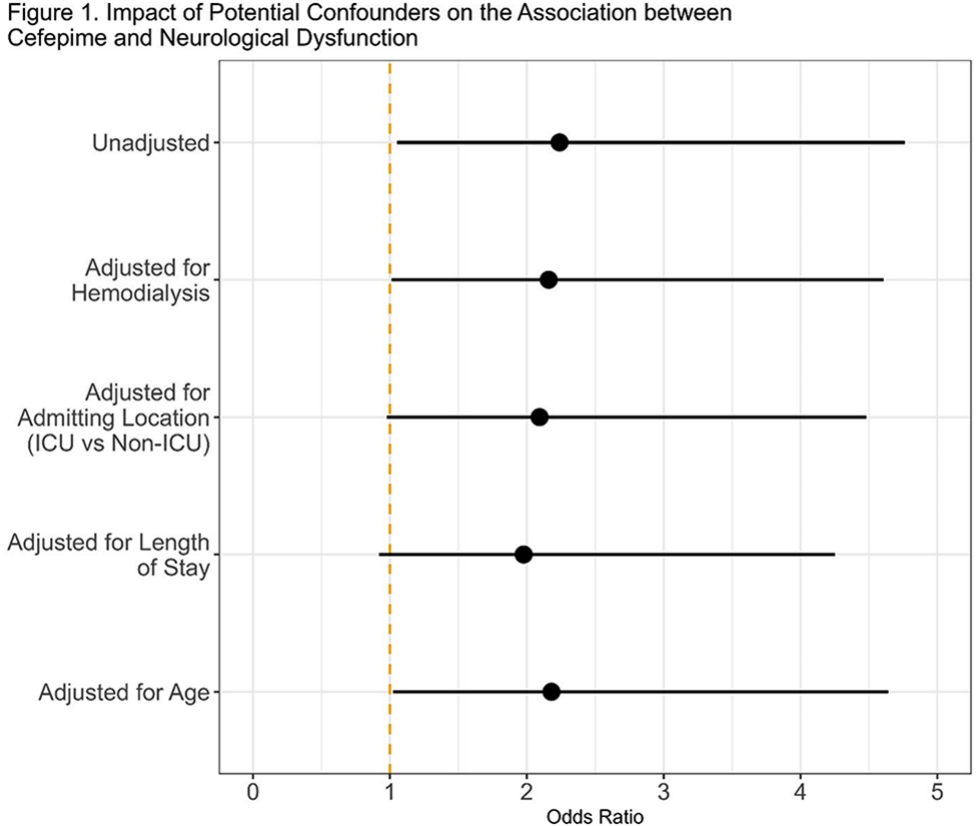

**Methods:**

This was a multicenter, retrospective, cohort study of inpatients at two hospitals in the Atrium Health Wake Forest Baptist system from Mar 2024 to Oct 2024. Patients ≥ 18 yrs old who received FEP or P/T on two or more consecutive calendar days were included. Only the first qualifying healthcare encounter during the study period was used for each patient. Patients with a preexisting diagnosis for ND, eg Alzheimer's disease, and those who received both FEP and P/T were excluded. ND was defined by incidence of new ICD10 code for seizure disorder, encephalopathy, delirium, agitation, altered mental status, coma, and/or confusion which was associated with the encounter when FEP or P/T was administered. Variables assessed as potential risk factors for ND included: patient characteristics (age, sex, race, BMI, comorbidities); presence of sepsis; hospital length of stay; admitting location (ICU vs non-ICU); hospital location; and antibiotic choice (FEP vs P/T).

**Results:**

2,642 patients were included in the analysis after excluding 1,461 patients for preexisting ND and 193 patients for receiving both antibiotics. 1,190 and 1,452 patients received FEP and P/T, respectively. Patient characteristics are presented in Table 1. 31 (1.2%) patients developed ND. Choice of FEP (1.68 vs 0.76%; OR 2.24, 95% CI 1.09-4.86, p=0.033) and longer length of stay (9 vs 6 days; OR 1.75, 95% CI 1.12-2.71, p=0.013) were the only factors associated with ND. Multivariable adjustments for possible confounders revealed no impact on the association between FEP and ND (Figure 1).

**Conclusion:**

Use of FEP is associated with ND as determined by new diagnoses (ICD10 codes). However, the overall rate of ND is low, and the risk of this adverse effect may not justify routine consideration when selecting empiric antibiotic therapy.

**Disclosures:**

All Authors: No reported disclosures

